# Interplay of population genetics and dynamics in the genetic control of mosquitoes

**DOI:** 10.1098/rsif.2013.1071

**Published:** 2014-04-06

**Authors:** Nina Alphey, Michael B. Bonsall

**Affiliations:** Mathematical Ecology Research Group, Department of Zoology, University of Oxford, South Parks Road, Oxford OX1 3PS, UK

**Keywords:** population genetics, population dynamics, genetic vector control, homing endonuclease gene

## Abstract

Some proposed genetics-based vector control methods aim to suppress or eliminate a mosquito population in a similar manner to the sterile insect technique. One approach under development in *Anopheles* mosquitoes uses homing endonuclease genes (HEGs)—selfish genetic elements (inherited at greater than Mendelian rate) that can spread rapidly through a population even if they reduce fitness. HEGs have potential to drive introduced traits through a population without large-scale sustained releases. The population genetics of HEG-based systems has been established using discrete-time mathematical models. However, several ecologically important aspects remain unexplored. We formulate a new continuous-time (overlapping generations) combined population dynamic and genetic model and apply it to a HEG that targets and knocks out a gene that is important for survival. We explore the effects of density dependence ranging from undercompensating to overcompensating larval competition, occurring before or after HEG fitness effects, and consider differences in competitive effect between genotypes (wild-type, heterozygotes and HEG homozygotes). We show that population outcomes—elimination, suppression or loss of the HEG—depend crucially on the interaction between these ecological aspects and genetics, and explain how the HEG fitness properties, the homing rate (drive) and the insect's life-history parameters influence those outcomes.

## 1. Introduction

Molecular biology tools are facilitating new genetics-based, species-specific methods of controlling insect pest populations, with the intention of improving efficacy over current methods and reducing adverse environmental consequences. In public health, the new technologies aim to reduce the burden of vector-borne diseases; key targets are the mosquito species *Anopheles gambiae* (major vector of human malaria, which causes 150–270 million cases and 0.6–1.6 million deaths annually [1,2]) and *Aedes aegypti* (principal vector of dengue virus, with *ca* 50–100 million infections and 18 000–19 000 deaths a year [3,4]). Transgenic approaches, involving the release of genetically modified (GM) mosquitoes into natural populations, include a broad class of population reduction methods aiming to suppress the numbers to a lower level or possibly local elimination, based on the principles of the sterile insect technique (SIT) [5]. Released GM mosquitoes mate with wild mosquitoes, potentially affecting the population genetics and population dynamics of the natural population, so ecological understanding is important if these vector control approaches are to improve human health [6,7].

Homing endonuclease genes (HEGs) are ‘selfish’ genes that can spread rapidly through populations even if they harm the host organism, because they are inherited at a higher than Mendelian rate [8]. HEGs exploit cellular repair mechanisms to copy themselves. A HEG encodes an endonuclease that recognizes and cuts a very short, specific DNA sequence (about 15–30 bp). The HEG sits inside its recognition sequence, so, in a heterozygous individual, the target sequence on the homologous chromosome is cut, and the cell's DNA repair machinery may use the HEG-bearing (HEG^+^) chromosome as a repair template, which results in conversion of a HEG heterozygote to a HEG homozygote. Engineered HEGs have potential to drive introduced traits, such as sterility or inability to transmit disease, through a mosquito population without needing large-scale sustained releases. Use of a meiosis-specific promoter to control the HEG allows heterozygous individuals to develop normally, but causes biased transmission of the HEG to their gametes. Two strategies are under development in *Anopheles* mosquitoes [9]. Releasing males carrying a HEG construct that targets a gene essential for female fertility could substantially reduce a target population of mosquitoes in similar manner to SIT. Males carrying an X-targeting HEG construct on their Y chromosome will produce a male-biased sex ratio, reducing both the number of mosquitoes (fewer females laying eggs) and disease transmission (fewer females biting). Some transgenic lines that were intended to target X-bearing sperm in this manner, actually created genetically sterile males (protein transferred to the zygote with the sperm resulted in damage to the maternal X chromosome, causing embryo lethality) [10], and those mosquitoes are in early stage trials [11]. As part of a broader programme of research on the ecology and genetics of insect control, here we formulate a population ecological and genetic model and apply it to the simple case of a HEG, present in both sexes, that targets a gene essential for survival, thereby causing pre-adult lethality.

The population genetics of HEG-based systems has been established using discrete-time mathematical models [12,13]. However, this can only form part of our understanding of this approach as a vector control method, as several ecologically important aspects remain unexplored. The goal is fewer mosquitoes, so population dynamics must be combined with the population genetics. Here, we address three key population dynamic issues: overlapping generations, density-dependent larval competition and the timing of the HEG's effect on survival relative to density-dependent mortality.

Little is really known about intra- or interspecific competition among most species of vector mosquitoes, however, there is growing evidence that competition gives rise to density-dependent survival through the larval stages [14–22]. Although it is not thought to be common in natural populations, overcompensatory competition has been demonstrated in a variety of insect species [23–26], including *Anopheles arabiensis* [25] and one of several possible model fits to *Ae. aegypti* field data [14,15]. Recent work [27], coupling a HEG population genetics model with simple dynamics, has shown that HEG-based vector control can potentially suppress or eliminate a mosquito population in timescales that are practical for disease control; combination with simple malaria epidemiology suggests that there is little scope to eliminate disease transmission without also eliminating the mosquito population. Those results were generated using a density function that imposed extra larval mortality that increased monotonically with larval density, and did not allow for the possibility of overcompensating larval competition. Among other aims, here we extend this finding to explore a wider range of mechanisms of density-dependent competition. In view of the lack of empirical evidence, we consider a broad range of strengths of density dependence to examine the effects.

Mathematical modelling of other genetic strategies predicts that late-acting genetic lethality is better at controlling populations that are restricted by density-dependent larval mortality [27–29]. We explore fully and test the hypothesis that HEG mortality acting after that competition should be more effective for population control than early-acting HEG mortality.

Mosquitoes do not exhibit synchronized discrete generations, but overlapping generations occur with all life stages (eggs, various instar larvae, pupae and adults) coexisting in natural habitats. Here, we formulate and analyse a continuous-time model to explore both mosquito population genetics and population dynamics. We adapt little-known work by Kostitzin [30], whose method applies Lotka–Volterra style competition equations (with linear competition based on the logistic equation) to different ‘groups’ (genotypes) rather than different species. We use a fixed time delay to represent density-dependent competition in immature stages, and extend Kostitzin's approach to incorporate a flexible two-parameter nonlinear intraspecific competition model which allows us to investigate the effects of under- or overcompensating density-dependent population regulation and to vary the competitive ability (as well as survival) between genotypes. We apply this set of delay differential equations to the release of mosquitoes bearing an early-acting or late-acting HEG that targets a gene important to survival, and investigate the interplay between population genetics and population dynamics. We provide a richer understanding of the population genetics and show that the potential efficacy of vector control depends crucially on the ecology (particularly population dynamic regulation) and the genetics.

## 2. Material and methods

We set out the state variables and parameters in table 1 and the full mathematical model in table 2. This is a deterministic model, assuming a panmictic (random mating) population with no genetic mutation or genetic drift. A fuller, more detailed derivation of the model is given in the electronic supplementary material, section Methods.

**Table 1.** Summary of symbols, state variables and parameters.

symbol	description	value simulated	reference and comments
*ww*, *Hw*, *HH*	genotypes: wild-type, heterozygous and homozygous HEG^+^	—	labelled *i* = 1,2,3; genotypes 1: *ww*, 2: *Hw*, 3: *HH*
*N_i_*(*t*)	number of adult mosquitoes of genotype *i*	—	state variables of the model
*N*(*t*)	total number of adult mosquitoes	—	
*ν_i_*	number of progeny (eggs) of each genotype arising from the various mating crosses among adults, incorporating the effects of homing during meiosis	—	see formulae in table 2
*τ*	time delay: generation time from egg to adult	16 days	duration of egg + larva + pupa stages (1 + 14 + 1) in *Anopheles gambiae* [27,31]
*μ*	adult mortality rate	0.123 per day	estimated for *A. gambiae* [27,32,33]
*r*	intrinsic *per capita* population growth rate (net of density-independent survival through immature stages)	1.096 per day	estimated as 1.096 (±0.0056) [27] from *Anopheles* data in [32]
*ρ*	per adult lifetime contribution of progeny to next adult generation	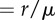	net of density-independent survival through immature stages
*b*	strength of density-dependent intraspecific competition	various	*b* > 1 represents overcompensating (scramble-like) competition among larvae, *b* < 1 undercompensating
*a*	density parameter, related to scale (at abundance 1/*a* the population experiences half of the maximum possible growth rate, due to density-dependent processes)	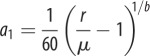	calculated to give natural equilibrium 60 mosquitoes per human host, based on estimate of 30 vector (female) *Anopheles* mosquitoes per host derived [27] from human biting rate data across Africa [34]
*θ*	(optional) factor altering relative competition coefficient for HEG^+^ homozygotes	various	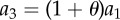 . Where *θ* > 0, larval competition affects all larvae at a smaller (lower density) scale, putting more competitive pressure on all genotypes. In single genotype populations, *HH* experience density-dependent effects at lower scale (1/*a*_3_) than wild-type larvae (1/*a*_1_); as a result the environment can support fewer transgenic larvae. *θ* < 0 represents the opposite
*ϕ*	(optional) dominance of HEG effect on competition coefficient in *Hw*	0 ≤ *ϕ* ≤ 1	*a*_2_ = (1 + *ϕθ*)*a*_1_. Heterozygotes may experience a weaker change in competitive performance (or none)
*g*	homing rate: the proportion of successful conversions of HEG^−^ to HEG^+^ in heterozygous individuals at meiosis	various 0 < *g* ≤ 1	homing rate approximately 0.6 achieved (range 0.56–0.7) for a synthetic HEG with a testis-specific promoter [35], and approx. 0.9 for an engineered HEG causing Y-chromosome drive (transmission to 88% of progeny embryos) [10]
*s*	relative fitness cost of the knockout	0 < *s* ≤ 1	0 (no penalty) to 1 (lethal)
*h*	dominance of that fitness cost	0 ≤ *h* ≤ 1	0 (fitness costs are recessive) to 1 (dominant)
*ψ_i_*	relative fitness parameters reflecting HEG mortality	—	*ψ*_1_ = 1, *ψ*_2_ = (1 − *hs*), *ψ*_3_ = (1 − *s*)
*L*	HEG load		see equations (3.3); the relative reduction in the population growth rate of the population in the presence of the HEG

**Table 2.** Model for genetic mosquito control strategy using a HEG affecting survival.

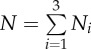
*ψ*_1_ = 1, *ψ*_2_ = (1 − *hs*), *ψ*_3_ = (1 − *s*)
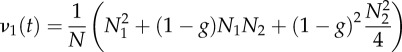
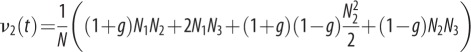
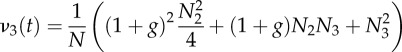
(1) early-acting HEG fitness cost 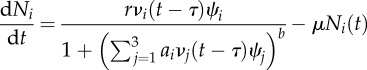 *i* = 1,2,3
(2) late-acting HEG fitness cost 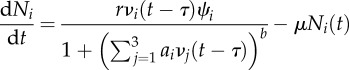 *i* = 1,2,3

*N_i_* denotes the number of adults of genotype *i* (*i* takes values 1: *ww*, 2: *Hw*, 3: *HH*, where *H* is HEG^+^ and *w* is HEG^−^ wild-type). These variables are real numbers (not integers), in effect assuming a large population, and are expressed in units of adult mosquitoes per host. Adult mortality occurs at rate *μ*, and intrinsic *per capita* growth rate *r* (net of density-independent mortality during immature stages) and *μ* are identical for all types. Population growth terms *rN_i_* are replaced by *rν_i_*, where the progeny arising *ν_i_* are functions that reflect mating crosses among the three genotypes [30], and incorporate the effects of homing. The homing rate *g* is the proportion of successful conversions of *w* (HEG^−^) to *H* (HEG^+^) in heterozygous individuals during meiosis. Density-dependent competition among larvae is reflected in the equations, with some simplifying assumptions, using a fixed time delay *τ* [36]. The composition and number of new adults emerging at time *t* depend on the number and type of adults that were mating and laying eggs at a previous time *t* − *τ*.

We formulate two versions of this model (table 2):

(1) ‘early-acting’ HEG fitness costs, where HEG mortality occurs after homing and before density-dependent competition effects—e.g. the knockout renders fertilized eggs non-viable; or
(2) ‘late-acting’ HEG fitness costs, where HEG mortality occurs after density-dependent competition and before mating—e.g. the target gene is essential for pupation.

Relative fitness parameters *ψ*_1_ = 1, *ψ*_2_ = (1−*hs*), *ψ*_3_ = (1−*s*) incorporate HEG-induced mortality, such that homozygotes suffer a fitness penalty (*s* > 0) as a result of the target gene being knocked out and, unless that knockout is recessive (*h* = 0), heterozygotes incur a partial fitness penalty (*hs* where 0 ≤ *h* < 1). For each genotype, the net population growth term is multiplied by the relative fitness *ψ_i_*. This applies to both early- and late-acting HEGs. However, early- and late-acting HEGs have a different impact on larval competition (see below), because of the timing of the induced mortality.

Bellows [23] examined several density-dependent competition functions of different forms and assessed their ability to represent 30 datasets exhibiting a range of intraspecific population dynamic behaviours. We adapt the nonlinear competition function that Bellows judged most flexible, based on that of Maynard Smith & Slatkin [37]:2.1
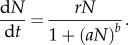


All genotypes experience the same strength of density-dependent intraspecific competition (which can range from contest to scramble by varying the value of coefficient *b*, with *b* > 1 denoting overcompensating scramble-like competition). The density-dependent scale parameters are either a single coefficient *a* or are genotype-specific *a_i_*. This allows us to explore the effects where the HEG can alter the competitive performance of transgenic larvae and not just the proportion surviving to reproductive maturity; a different value of *a_i_* could be thought of as representing different resource needs (perhaps transgenic individuals are smaller or have different metabolism from wild-type), and therefore altering the scale at which density-dependent regulation acts (and the number of that genotype that the habitat is capable of supporting). Mortality from a late-acting HEG occurs after the larvae have participated in density-dependent competition, so it does not appear in the competition term, i.e. the denominator of the fraction. An early-acting HEG will already have reduced the numbers of larvae, so the survivors suffer less competitive conditions, with the relative fitnesses (*ψ_i_*) reducing the effect of competition.

The gametic frequency of the HEG at time, *t*, is determined after homing: we label the frequencies of *H* HEG^+^ (*q*) and *w* HEG^−^ (*p*) sperm and eggs produced by adults at time *t*, where *p*(*t*) + *q*(*t*) = 1. GM insects are released into the population at time *t* = 0; the gametic frequency at that time is denoted by *q*_0_. Mortality of those released adults reduces the gametic frequency to *q_*τ*_* by the time the first transgenic progeny emerge as adults. We numerically simulated a one-off release of a number of adults equal to 25% of the equilibrium of the natural population (0.25N*), all of them heterozygotes (*Hw*), giving an instantaneous HEG allele frequency of 0.1. (For any given homing rate *g*, the gametic frequencies *q*_0_ and *q_*τ*_* can then be calculated; for our default homing rate value, *g* = 0.8, gametic frequencies are *q*_0_ = 0.18 and *q_*τ*_* = 0.0304.)

## 3. Results

### 3.1. Population genetics

Our population genetic outcomes are consistent with previous work [13], albeit with one new element resulting from taking account of the biological time lag (*q_*τ*_* substitutes for *q*_0_ where gametic frequency is necessary to determine the outcome). Gametic frequencies *q* = 0 (HEG not present or extinct) and *q* = 1 (HEG reaches fixation) are always equilibrium solutions. An internal equilibrium q* (0 < q* < 1) exists if and only if a specified relationship between the genetic properties of the HEG (relative fitness costs of gene disruption *s*, dominance of fitness costs *h* and homing rate *g*) holds true. Where it exists3.1
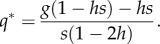


By boosting allele transmission rates, homing confers an advantage on the HEG. Homing occurs only in a heterozygous individual, during meiosis, where it converts (at rate *g*) a HEG^−^ (*w*) gamete to HEG^+^ (*H*). This results in reduced fitness (survival) of the resulting progeny. If the other parent contributes a *w* gamete, then the zygote that would have been wild-type (*ww*, no fitness penalty) becomes heterozygous (*Hw*, with lower relative fitness *ψ*_2_ = (1 − *hs*)). This incurs a selection disadvantage relative to the fitness of the heterozygous parent in which homing occurred3.2a
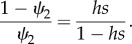


Similarly, if the other parent contributes an *H* gamete, then a zygote that would have been heterozygous (*Hw*, with fitness *ψ*_2_ = (1 − *hs*)) is instead homozygous (*HH*, with lower fitness *ψ*_3_ = (1 − *s*)) and the selection disadvantage relative to the fitness of the *Hw* parent in which homing occurred is3.2b
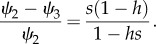


**Figure 1.**
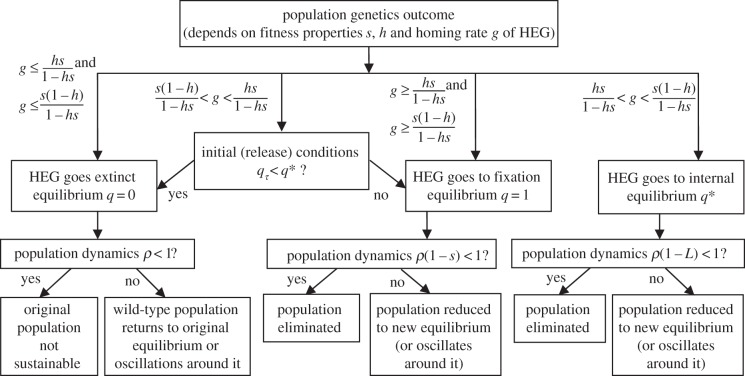
Overview of outcomes. This summarizes the way in which mosquito population genetics and dynamics interact. The population genetic outcome depends on the fitness properties of the HEG (cost *s*, and its dominance *h*) and the homing rate (*g*). The population dynamic implications depend on the relationship between the life-history parameters (*ρ* = *r/μ* lifetime progeny net of density-independent juvenile mortality, and *τ* egg-to-adult generation time) and the HEG parameters, particularly the HEG load (0 for *ww*, *s* in *HH* population, or *L* (equation (3.3)) at mixed genotype equilibrium).

In a synthetic HEG, intended to spread through a vector population, the engineered gene disruption effect would preferably be nearer recessive than dominant (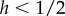). If so, then heterozygote fitness would be closer to that of wild-type than of *HH* homozygotes, and equation (3.2*a*) would be smaller than (3.2*b*). Otherwise, the converse is true. The population genetic outcomes, which are summarized in the upper part of figure 1, are a consequence of the relative weight of these forces and so are dependent on the relationships between the homing rate *g* and the phenotypic (fitness) properties of the HEG (*s*, *h*).

**Figure 2.**
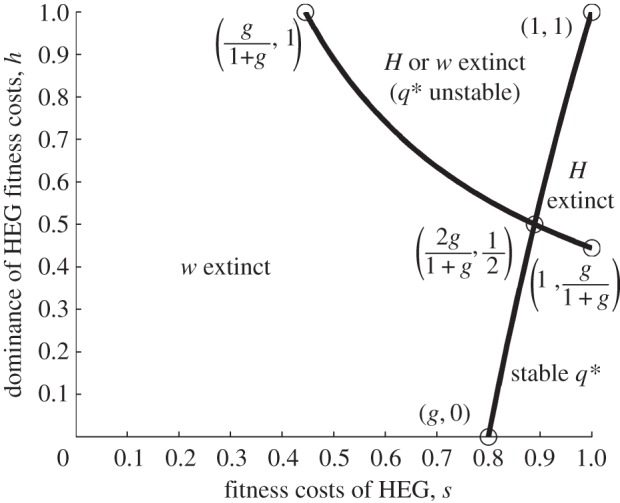
Population genetic outcomes relationship with *s* and *h.* The population genetic outcome depends on the phenotypic fitness properties of the HEG (*s*, *h*) and the homing rate (*g*). The graph shows the regions of parameter space defined by *s* (the HEG fitness penalty) and *h* (the dominance of that fitness cost), plotted for *g* = 0.8.

Figure 2 illustrates the four regions, in terms of *s* and *h*, where each of these population genetic outcomes occurs. If the drive advantage (*g*) is lower than both the selection disadvantages (equations (3.2*a*) and (3.2*b*)), then the HEG will go extinct, i.e. to gametic equilibrium *q* = 0. In that case, *q* = 1 is unstable, so any introduction of wild-type alleles into a HEG^+^ homozygote population should eventually remove the HEG. If the genetic drive (*g*) is high enough that it outweighs both causes of reduced fitness (equations (3.2*a*) and (3.2*b*)), then the HEG will spread to fixation; the gametic equilibrium is *q* = 1 (*q* = 0 is unstable, so the HEG will always invade even from very low levels). Assuming low dominance of fitness effects (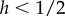), an intermediate drive (*g*) can favour *Hw* individuals (equation (3.2*a*)) but be insufficient to outweigh the selection against *HH* genotypes (3.2*b*), and these balancing forces drive the population to a stable internal equilibrium consisting of all viable genotypes. In that case, the equilibrium gametic frequency of the HEG is given by q* (equation (3.1)); either allele can invade from rare (*q* = 1 and *q* = 0 are unstable). If the HEG is strongly deleterious to heterozygotes (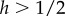), then an intermediate genetic drive (*g*) produces opposing selective forces that are frequency dependent. If the *H* allele is predominant in the population (*q_*τ*_* > q*), then the HEG will be driven to fixation, because the drive (*g*) outweighs the fitness effect of replacing *Hw* progeny with *HH* (equation (3.2*b*)), whereas if the HEG is relatively rare (*q_*τ*_* < q*), then it will become extinct, because homing does not overcome the disadvantage of replacing *ww* with *Hw* (equation (3.2*a*)). In those circumstances, neither allele can invade from rare (q* is unstable, *q* = 1 and *q* = 0 are stable).

Note that the gametic equilibrium reached is independent of initial gametic frequencies *q*_0_ and *q_*τ*_* except for that last case, i.e. a HEG that has strong fitness effects (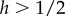) and an intermediate homing rate (the relevant inequalities are satisfied with moderate-to-high fitness cost, *s*; figure 2 and the electronic supplementary material, section Population genetics outcomes). In comparison, in all cases, the initial gametic frequency does affect the time taken to reach the predicted equilibrium frequency.

One key feature of using a continuous-time framework rather than a discrete generation model is that it takes account of the biological time lag from egg to adult emergence. In the period from release of HEG-bearing insects (*t* = 0) to the time when the first transgenic progeny emerge as adults (*t* = *τ*), the released GM insects reduce in number owing to mortality, but the wild-type adults are replenished by emergence of existing larvae. Consequently, in that period, the HEG gametic frequency is diluted from its instantaneous value at release to a frequency that may be significantly lower. With our default parameter values, gametic frequencies fall from *q*_0_ = 0.18 to *q_*τ*_* = 0.0304. In circumstances where the gametic frequency determines the population genetic outcome, it is very likely that the HEG allele will be sufficiently rare (*q_*τ*_* < q*) that the HEG will be lost from the population rather than driven to fixation. The quantity of insects released would have to be substantially higher to mitigate that effect. (See the electronic supplementary material, section Population genetics outcomes.)

The ‘HEG load’ (analogous to genetic load in classical population genetics) has been defined as the relative reduction in the population growth rate of the population in the presence of the HEG [13]. The HEG load in a wild-type population is zero, in a pure *HH* population it is *s*, and at the mixed genotype equilibrium q* the HEG load *L* is3.3a
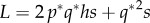
which, alternatively, can be expressed as3.3b
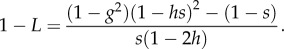
At the mixed genotype equilibrium, the HEG load is the sum of fitness penalties weighted by their genotype frequency (equation (3.3*a*)) and is influenced by the homing rate as well as the relative fitnesses of HEG homozygotes (1 − *s*) and heterozygotes (1 − *hs*) (equation (3.3*b*)).

### 3.2. Population dynamics

The natural wild-type population (time-delayed version of equation (2.1)) has the following equilibrium3.4
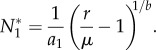


**Figure 3.**
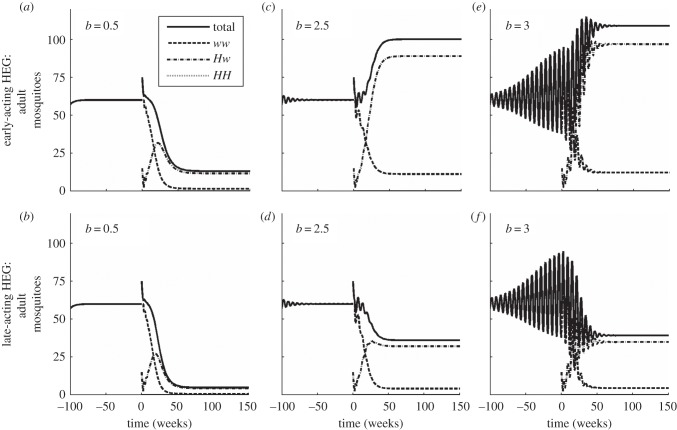
Dynamical behaviour. The HEG knockout is recessive lethal (*s* = 1, *h* = 0), homing rate *g* = 0.8. Heterozygous insects are released at time *t* = 0, 15 per host (i.e. + 25% of the natural population equilibrium 60 per host). The number of adults over time is shown for each genotype (*ww*: dashed line, *Hw*: dashed-dotted line, *HH*: dotted line, here always zero) and in total (solid line). Strength of density dependence *b* is 0.5 (weak, undercompensating, *a*,*b*), 2.5 (underdamped before release, *c*,*d*), or 3 (overcompensating, oscillating before release, *e*,*f*), the HEG is early-acting (*a,c,e*) or late-acting (*b,d,f*). See electronic supplementary material, figure S2 for further *b* = 1.097 and *b* = 5. In all figures and described results, the equilibrium size of the natural population remains comparable across different values of *b* (the density scale parameter *a*_1_ is calculated from N* = 60 and *b* for each panel). In all population dynamics figures, life-history parameter values are estimated for *Anopheles gambiae* (table 1): time lag *τ* = 16 days, adult mortality rate *μ* = 0.123 per day, net population growth rate *r* = 1.096 per day.

We assume that *r* > *μ* (otherwise, the wild-type population would naturally die out). We fix this natural equilibrium at 60 adult mosquitoes per host (table 1), which determines our value of *a*_1_ for every given *b*. It is useful to define 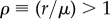, *per capita* lifetime reproductive potential. Our simulations and quantified analytical results are based on life-history parameter values drawn from the literature (table 1). We simulate and analyse results for several values of *b*, covering situations where the natural population dynamics are stable and overdamped (*b* < 1.097), or are stable with damped oscillations to the equilibrium (1.097 < *b* < 2.851), or exhibit natural oscillations about the equilibrium value (*b* > 2.851) (the electronic supplementary material, section Population dynamics: stability). These pre-release dynamics can be seen in figure 3, in the period *t* < 0. For an unstable population (highest values of *b*), we calculate an average population size 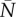 over the last five simulated cycles of oscillations, using the methods of Armstrong & McGehee [38]. The average size of an oscillating population is larger than the hypothetical steady state; the stronger the density dependence, the greater the amount by which 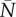 exceeds N*.

The effect of releasing HEG-bearing insects into the population is to introduce a HEG load. If the HEG spreads, then this reduces the reproductive ability of the population (in effect, a scaling factor is applied to *ρ*). Where the population is suppressed but not eliminated, this effect is more likely to give stable dynamics at the post-release equilibrium, as the stability boundaries correspond to higher values of *b*, the strength of density-dependent competition (the electronic supplementary material, section Population dynamics: stability). If the HEG goes extinct, then the population reverts to its natural equilibrium (equation (3.4)) or oscillatory behaviour. To predict these outcomes and, in particular, to ascertain the properties that are expected to lead to local elimination of the vector population and thereby eliminate disease transmission, it is necessary to explore the combined effects of population genetics and population dynamics.

### 3.3. Population dynamics combined with genetics

If the HEG's genetic properties allow it to spread through the vector population to some extent, then it disrupts the target gene and reduces survival. If a sufficiently high HEG load can be imposed, then the population can be locally eliminated. A less effective HEG load will suppress the population to a lower abundance which may (or may not) be significant. These effects can be achieved in timescales that are potentially useful for disease control. (See figure 3, illustrating a recessive lethal knockout.)

As expected, the late-acting version has a lower post-release equilibrium than early-acting (figure 3 compare bottom row with top). An early-acting HEG can actually increase the population (figure 3*c,e*). By killing eggs or early instars, the early-acting HEG system creates a lower-density environment in which unaffected individuals and survivors will compete, and with overcompensating (scramble-like) competition that population rebounds to a higher abundance. This effect is similar to that already known for classical and genetics-based SIT [39,40].

For an overview of results, see figure 1. Population elimination 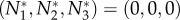 is an equilibrium for both early- and late-acting HEG constructs. It is achieved when the net population growth rate *ρ* multiplied by 1 – HEG load is below unity, otherwise the population will persist. Where the HEG properties are such that the HEG will go to fixation, the equilibrium is3.5a
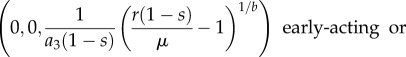
3.5b
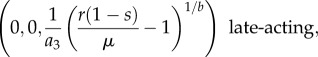
where *ρ*(1 − *s*) > 1; otherwise, a population of HEG homozygotes would die out. Where a mixed genotype population occurs, and *ρ*(1 − *L*) > 1, the equilibria satisfy the following (q* as per equation (3.1), p* = 1 − q*, and *L* per equation (3.3)).3.6a
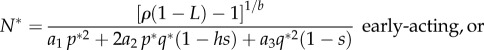
3.6b
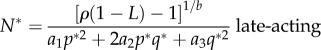
3.6c
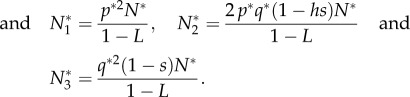
Equations (3.5*a,b*) and (3.6*a,b*) confirm that any persistent late-acting HEG achieves a lower population equilibrium than the equivalent early-acting HEG (the electronic supplementary material, section Population dynamic outcomes).

If density-dependent competition is very strong (high *b*), then the population will oscillate around the relevant equilibrium, with average value (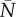) higher than that steady-state value (N*). A late-acting HEG achieves a lower average abundance than the equivalent early-acting HEG. The stability boundaries in a HEG-bearing population occur at higher values of *b* than for the natural population (the electronic supplementary material, section Population dynamics: stability). A population suppressed by HEG vector control is very likely to exhibit stable dynamics (e.g. figure 3 and the electronic supplementary material, table S2). The greater the HEG load imposed, the more stable the system is, and the more resilient it is to small perturbations.

**Figure 4.**
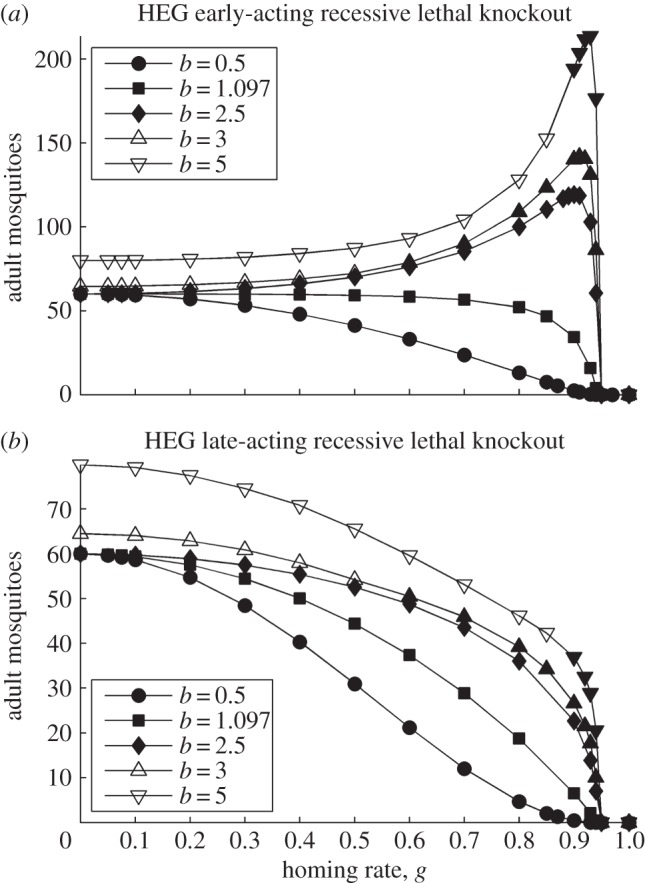
Effects of homing rate on population size. The population size is shown, the equilibrium N* where stable (filled symbols) or the average 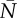 where oscillating (open symbols), for the spectrum of values of homing rate 0 ≤ *g* ≤ 1 (*g* = 0 results in wild-type population). The HEG is recessive lethal (*s* = 1, *h* = 0), early-acting (*a*) or late-acting (*b*). Strength of density dependence *b* is 0.5, 1.097, 2.5, 3 or 5 (key in *a*). Pre-release equilibrium N* = 60 per host for all values of *b*; in unstable natural populations, the pre-release average 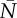 is higher than 60 (the same value as with *g* = 0).

In the mixed genotype case, increasing the homing rate *g* increases the HEG load (inspect equation (3.3*b*)) and so generally decreases the post-release population equilibrium (N*, equations (3.6)) and, if unstable (very high values of *b*), decreases the average density (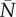). The exception is an early-acting HEG in a population with overcompensating density dependence (*b* > 1), where the HEG ‘load’ benefits the larval population, increasing the average adult abundance. These findings are illustrated for recessive lethal knockouts (*s* = 1, *h* = 0; figure 4). A recessive lethal HEG is always driven to an intermediate equilibrium frequency q* = *g* (equation (3.1)), whatever the homing rate (equation (3.2*a*) equals 0 and equation (3.2*b*) equals 1), and HEG load *L* = *g*^2^ (equation (3.3*b*)). In this case, the homing rate above which *ρ*(1 − *L*) < 1 and the population is eliminated is3.7
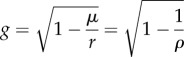

With our parameter values (table 1), this threshold value for a recessive lethal knockout is *g* = 0.942.

**Figure 5.**
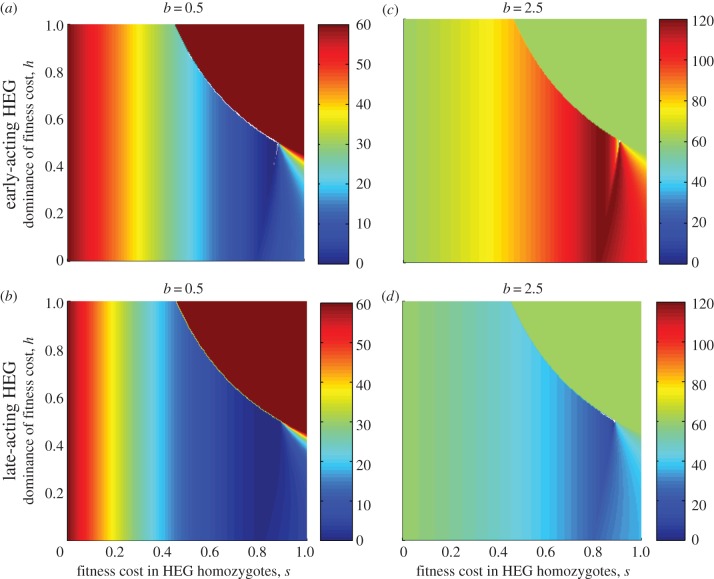
Effects of fitness properties due to gene disruption by the HEG on population size. Homing rate *g* = 0.8. All genotypes compete equally. These surface plots show the equilibrium N* (indicated by colour scales), for the spectrum of values 0 < *s* ≤ 1 and 0 ≤ *h* ≤ 1, following release of heterozygotes *N*_2_(0) equal to 0.25 times the natural population equilibrium. Strength of density dependence *b* is 0.5 (weak, undercompensating; *a,b*) or 2.5 (strong, overcompensating; *c,d*), the HEG is early-acting (*a,c*) or late-acting (*b,d*).

The HEG fitness properties, (*s*, *h*), affect the equilibrium (N* where stable; figure 5) or average (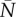 where unstable) population density, through the four underlying population genetics cases (figures 1 and 2). With homing rate *g* = 0.8, for most combinations of *s*, *h*, the HEG spreads to fixation, and the equilibrium density (equations (3.5)) decreases as the HEG-induced mortality *s* increases (figure 5*a*,*b*,*d*). With an early-acting HEG, a population under strong density-dependent competition rebounds to higher than natural densities (figure 5*c*). Where fitness cost (*s*) is high and nearer recessive than dominant (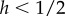), the population reaches a mixed genotype equilibrium giving intermediate suppression (figure 5, lower right of each panel *a*,*b*,*d*). Where *s* and *h* are both high (figure 5, upper right of each panel *a*–*d*), the HEG is driven extinct, and the population returns to its natural equilibrium. For much of that region (except a triangular area at the high *s* end; figure 2), the outcome might be altered by releasing far more transgenic insects, so that the HEG is sufficiently common to tip the balance to HEG fixation instead of extinction. Even with this high homing rate (0.8) and a fairly large release (augmenting the original population by one-quarter), there are few combinations of *s* and *h* that suppress the population to a level at or close to local elimination. These combinations occur when 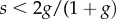 and 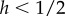, close to the point 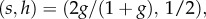 where the four regions of population genetic outcomes intersect (figure 2); the HEG is at fixation.

**Figure 6.**
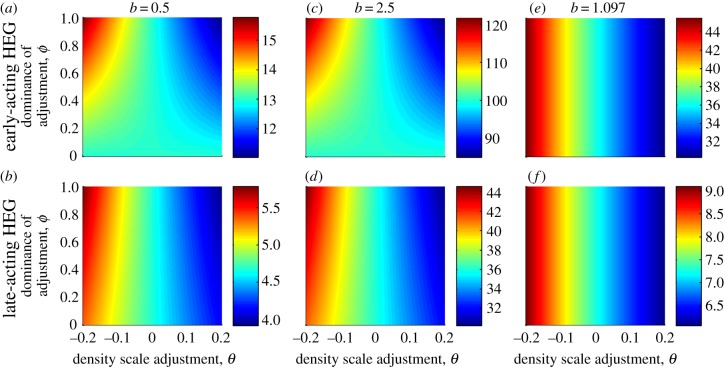
Effects of the HEG altering the scale at which density-dependent competition acts on population size. The post-release equilibrium N* is shown (indicated by colour scales), for a range of values of *θ*(−0.2 to +0.2), which alters the density scale parameter *a*_3_, and the spectrum of dominance of that effects *ϕ* (from 0 recessive to 1 dominant), which alters *a*_2_. The HEG has homing rate *g* = 0.8 and is early-acting (*a,c,e*) or late-acting (*b,d,f*). Strength of density dependence *b* is 0.5 (*a,b*), 2.5 (*c,d*) or 1.097 (*e,f*). The HEG knockout is recessive lethal (*s* = 1, *h* = 0), except in (*e*) and (*f*) where *s* = 0.8, *h* = 0.1 which results in the HEG going to fixation so then *a*_2_ is irrelevant (similar pattern for any *b*).

The effect of differential competition among genotypes is explored by varying *θ* (> or <0) to alter the relative competition effect of *HH* genotypes and *ϕ*, the dominance of that impact (table 1; *a*_3_ = (1 + *θ*)*a*_1_ and *a*_2_ = (1 + *ϕθ*)*a*_1_). This changes the equilibrium N* (and the average 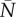, if unstable) in a suppressed population. Relative to the result with equal competition, the effect of unequal competitive effects is to multiply the equilibria by:3.8a
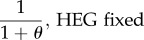
3.8b
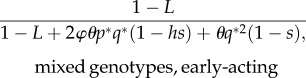
and3.8c
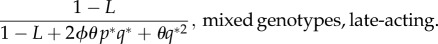
If the effect is dominant (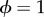), then the expressions (3.8*b*) and (3.8*c*) collapse to 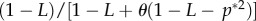 and 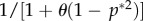, respectively. If transgenic insects require greater resources and therefore exert competitive pressure at lower scales (*θ* > 0), then the suppressed population will settle to a lower density than if all genotypes had competed equally (and the opposite for *θ* < 0). The stronger the dominance of this alteration to competitive effect (higher *ϕ*), the larger the impact on population size (illustrated in figure 6).

We explored a comprehensive range of values of *s*, *h*, *g*, *b* and *a_i_*. Our findings are also robust to changes in *τ*, *r* and *μ* (the electronic supplementary material, section Population dynamics outcomes).

## 4. Discussion

We have devised a novel mathematical modelling method for combining population genetics and population dynamics in a continuous-time framework with density-dependent competition. We applied it to a genetic vector control strategy using HEGs to drive a gene knockout through a population, inspired by a system that is in development and progressing towards implementation. We have shown that across a range of possible parameter sets, the outcome is generally more likely to be population suppression than to be local elimination. There have been few ecological studies of the population dynamics of *A. gambiae* mosquitoes, of which very few were conducted in semi-natural conditions [17,41]. Given our poor understanding of population regulation in malaria vectors, our results highlight the variation in possible outcomes of HEG vector control under different assumptions about the strength of density-dependent larval competition.

The influences on population genetics behaviour can be separated out between the HEG's fitness properties (the penalty to survival, *s*, and the dominance of that fitness cost *h*) and the homing rate *g*. Engineering a synthetic HEG targeting a gene that is important for survival and creating transgenic insect lines with that HEG positioned within its recognition site is challenging, and fitness properties, homing rates and regulation of gene expression cannot be precisely controlled [42]. A homing rate *g* > 0.942 is needed for a recessive lethal knockout to locally eliminate a population (equation (3.7)). Given the homing rates achieved in experimental strains, 0.56 to approximately 0.9 [10,35], such a high rate presents a challenging target. It has been proposed that to achieve a high enough HEG load in practice with realistic homing rates, multiple HEGs could be used, each targeting a different gene, with independent fitness effects across loci [9]. More sophisticated HEG systems are being developed for field use, targeting female fertility or imposing a male bias, and it is intended to combine multiple copies in a released strain to increase the likelihood of control success.

If the HEG persists within the population (at fixation or some intermediate frequency), then the relationship between the genetic parameters (*s*, *h* and *g* within HEG load *L*, or simply the HEG load *s* at fixation) and the life-history traits (*ρ*) determine whether the mosquito population is locally eliminated or merely suppressed. The higher the reproductive potential of the population (*ρ*), the greater the HEG load needed to overcome it. For example, the conditions for population elimination are 1 − *s* > 1/*ρ* for a population in which the HEG goes to fixation, or 
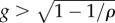
 for a recessive lethal knockout (equation (3.7)). Even in these simpler cases, it is necessary to understand the population dynamics and not just the population genetics, to predict whether local elimination is feasible. Close to thresholds, elimination can take a long time to achieve.

Our model is restricted to consider only a single value of *b*; all genotypes experience the same strength of density-dependent competition. This is a mathematical consequence of using nonlinear population dynamic models of this kind (electronic supplementary material, section Methods). We have modelled a wide range of strengths from very weak (undercompensating competition with low *b*) to very strong (overcompensating with high *b*), to explore the range of possible effects. Our illustrations present results generated with some parameter values for *b* that may be regarded as implausibly high or low, not to suggest that such extremes are to be expected, but for ease of visibly discerning the nature of the effects identified across the spectrum of strength of density dependence.

One novel consideration in our study is altering the competitive impact of larvae bearing one or two copies of the HEG transgene. The density scale parameter in this model is manifested as a competitive effect (a change affects larvae of all genotypes) rather than as a competitive response. If the parameter is higher for HEG homozygotes than wild-type insects (*a*_3_ > *a*_1_), then *HH* larvae contribute greater competitive pressure on all genotypes. This might plausibly occur, for example, if *HH* individuals process food less efficiently and need to consume more nutrients during development, reducing the nutritional resources available to larvae of all types. As the transgenic insects have greater resource utilization needs, the environment would be able support fewer of them. By contrast, an example of different competitive response might be less ability to use an alternative food resource to ameliorate some of the pressure. More sophisticated models would be required to tease out such effect/response distinctions. In our model, the density scale parameters do not affect whether a population is eliminated or persists following HEG release, nor the equilibrium mix of genotypes. They do affect the population size, by changing the scale at which density-dependent effects have significant impact, and so would affect the disease transmission capability of the vector population following release.

Where the population can be suppressed but not eliminated, as is the case across most of the parameter space for properties of the HEG even with our generous homing rate 0.8 (figure 5), a crucial question is whether the new equilibrium is below the entomological threshold necessary to sustain disease transmission. As estimates of the basic reproduction number of malaria (*R*_0_, or *Z*_0_) across Africa vary from near 1 to over 3000 [43], broad general assertions are inappropriate, but some patterns can be observed. If the wild population is under strong density-dependent competition with oscillatory dynamics, then HEG suppression of the population would have a dual effect on population size, by both inducing stable dynamics (the steady-state N* is lower than the average 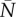) and reducing the equilibrium size, potentially increasing the chances of a successful epidemiological outcome. A HEG that affects both survival and competitive ability (increased density scale parameter) can be more effective at population suppression than a HEG that impacts on survival alone, which also enhances the potential for disease reduction. Depending on the extent of suppression, stochastic effects (which are not included in our deterministic framework) could become relevant, as the mosquito population could be eliminated at low numbers. In principle, a deterministically persistent HEG might become extinct in a small stochastic population owing to genetic drift.

As we and others have noted [15,16], there is a lack of data about density-dependent competition in the field for mosquito disease vector species. The few experiments published have almost all studied container-dwelling *Aedes* mosquitoes, where overcompensating competition is thought to be possible, and almost nothing is known about *Anopheles* mosquitoes (an exception being ref. [17]), which will be critical for applying genetic control tools to malaria vectors. Methods have been demonstrated for studying natural container-breeding populations in natural settings (without artificial addition of water or food materials), varying mosquito densities across natural ranges [21,22]; more of such manipulative ecological studies to determine how density dependence acts and to test for compensatory effects of added mortality would have long-term value, especially if they can quantify the form and shape of density dependence functions for modelling genetic vector control. This is one of several challenges in mosquito ecology, another is estimating the intrinsic rate of population increase. Addressing these ecological challenges could make a real contribution to developing, regulating and implementing feasible vector control methods. For example, regulators might be concerned at the theoretical prospect of early-acting (but not late-acting) HEG releases possibly increasing a vector population that is regulated by overcompensating competition, although there is no evidence of overcompensating density-dependent larval competition in *A. gambiae* in the field and the potential risk might be unfounded. Incorporating stochasticity and spatial and temporal heterogeneity into models, might also dampen the propensity for strong oscillations in population dynamics.

Although we applied our method to a particular application of HEGs to mosquito control, this approach has much broader relevance. It can be extended to other HEG strategies (for example, a HEG targeting female fertility can be modelled by expanding the system with separate equations for males and females) and, in principle, to other genetic vector control strategies. Our framework could be expanded to include a second insect species, with intraspecific and interspecific competition following the same functional form (with different resource utilization) [44]. Further aspects of mosquito ecology could be incorporated, such as seasonal fluctuations in population size or extension to structured populations with limited interbreeding. Entomological models could be coupled with epidemiological models to explore the potential for alleviating human disease. This would be necessary for assessing strategies that aim to propagate a genetic refractoriness to disease transmission through the vector population. Extension to include a health economic analysis could be used to assess the consequent reduction in disease burden [45].

Previous models of selfish genetic elements used discrete-time population genetic frameworks to deduce mathematical conditions for invasion of the novel element primarily in terms of biased gene conversion (transmission) and reduced survival (fitness) [9,12,13,46]. Those papers that explicitly considered vector population control applications explored outcomes in terms of allele or gametic frequencies, either with no direct modelling of the effect on population size [9,12,13], or with fairly simple population dynamics [27]. Our study investigated the effects of the opposing forces of drive and natural selection in a richer population dynamic framework that is able to represent a wide range of density-dependent larval competition dynamics, the relative timing of key biological processes, and fitness effects that alter competitive ability as well as reduce survival. Our key novel findings include that: the HEG gametic frequency is significantly diluted between the release of insects and the adult emergence of their first transgenic progeny, during which time further wild-type larvae emerge as adults (with practical implications for how many insects to release); a persistent HEG has a stabilizing effect on population dynamics, which could further suppress the mean vector abundance (for more effective disease control); an early-acting HEG performs worse than a late-acting HEG and would be counterproductive in the presence of overcompensating density-dependent larval competition; a HEG altering competitive effect as well as survival can give more effective vector control. We have shown that mosquito control outcomes depend on the interplay between genetics and ecology (here focusing on overlapping generations, larval competition and the maturation time delay from egg to adult) and argue that both aspects need to be appropriately considered when assessing the potential effectiveness of genetic vector control methods.

## Data accessibility

This project generated computer code (in Matlab) and simulation data. For access to the scripts or data files, see our website http://merg.zoo.ox.ac.uk and contact the authors.
